# Protein Misfolding as an Underlying Molecular Defect in Mucopolysaccharidosis III Type C

**DOI:** 10.1371/journal.pone.0007434

**Published:** 2009-10-13

**Authors:** Matthew Feldhammer, Stéphanie Durand, Alexey V. Pshezhetsky

**Affiliations:** 1 Department of Medical Genetics, CHU Sainte-Justine University of Montreal, Montreal, Canada; 2 Department of Biochemistry, University of Montreal, Montreal, Canada; 3 Department of Pediatrics, University of Montreal, Montreal, Canada; 4 Department of Anatomy and Cell Biology, Faculty of Medicine, McGill University, Montreal, Canada; National Institutes of Health, United States of America

## Abstract

Mucopolysaccharidosis type IIIC or Sanfilippo syndrome type C (MPS IIIC, MIM #252930) is an autosomal recessive disorder caused by deficiency of the lysosomal membrane enzyme, heparan sulfate acetyl-CoA: α-glucosaminide N-acetyltransferase (HGSNAT, EC 2.3.1.78), which catalyses transmembrane acetylation of the terminal glucosamine residues of heparan sulfate prior to their hydrolysis by α-N-acetylglucosaminidase. Lysosomal storage of undegraded heparan sulfate in the cells of affected patients leads to neuronal death causing neurodegeneration and is accompanied by mild visceral and skeletal abnormalities, including coarse facies and joint stiffness. Surprisingly, the majority of MPS IIIC patients carrying missense mutations are as severely affected as those with splicing errors, frame shifts or nonsense mutations resulting in the complete absence of HGSNAT protein.

In order to understand the effects of the missense mutations in HGSNAT on its enzymatic activity and biogenesis, we have expressed 21 mutant proteins in cultured human fibroblasts and COS-7 cells and studied their folding, targeting and activity. We found that 17 of the 21 missense mutations in HGSNAT caused misfolding of the enzyme, which is abnormally glycosylated and not targeted to the lysosome, but retained in the endoplasmic reticulum. The other 4 mutants represented rare polymorphisms which had no effect on the activity, processing and targeting of the enzyme. Treatment of patient cells with a competitive HGSNAT inhibitor, glucosamine, partially rescued several of the expressed mutants.

Altogether our data provide an explanation for the severity of MPS IIIC and suggest that search for pharmaceutical chaperones can in the future result in therapeutic options for this disease.

## Introduction

Mucopolysaccharidosis III (also called Sanfilippo syndrome) is an autosomal recessive disease caused by lysosomal accumulation of heparan sulfate [reviewed in 1] and includes four allelic subtypes caused by the genetic deficiencies of heparan N-sulfatase (MPS III type A; MIM #252900), α-N-acetylglucosaminidase (MPS III type B; MIM #252920), heparan sulfate acetyl-CoA: α-glucosaminide N-acetyltransferase or HGSNAT (MPS III type C; MIM #252930), and N-acetylglucosamine 6-sulfatase (MPS III type D; MIM #252940). The majority of MPS IIIC patients have severe clinical manifestations with onset in infancy or early childhood. They rapidly develop progressive and severe neurological deterioration causing hyperactivity, sleep disorders, loss of speech accompanied by behavioral abnormalities, neuropsychiatric problems, mental retardation, hearing loss, and visceral manifestations, such as mild hepatomegaly, mild dysostosis multiplex, mild coarse facies, and hypertrichosis [2], [3]. A majority of patients experience severe mental retardation and die before adulthood but some survive to the fourth decade with progressive dementia and retinitis pigmentosa [1], [3]. In the very rare MPS IIIC patients with the onset of symptoms in adulthood the disease progression was similar in severity and time course to the forms with onset in childhood [4]. The birth prevalence of MPS IIIC in Australia, Portugal and the Netherlands was estimated at 0.07, 0.12 and 0.21 per 100,000, respectively [5], [6], [7].

Although from the moment of discovery MPS IIIC was recognized as a deficiency of an enzyme that transfers an acetyl group from cytoplasmically derived acetyl-CoA to terminal N-glucosamine residues of heparan sulfate within the lysosomes [8]–[10], the molecular defects causing the disease have not been characterized for almost three decades because the identification and cloning of HGSNAT has been hampered by low tissue content and instability of the enzyme. Recently our group and others cloned the gene coding for HGSNAT [11], [12] which paved the way for characterization of the molecular defects in MPS IIIC patients. To date, 54 HGSNAT sequence variants have been identified including 13 splice-site mutations, 11 insertions and deletions causing frame shifts and premature termination of translation, 8 nonsense, 18 missense mutations and 4 polymorphisms [reviewed in 13, http://chromium.liacs.nl/LOVD2/home.php?select_db = HGSNAT], While the HGSNAT transcripts with abnormal splicing, frame shifts and premature stop codons are rapidly degraded via the nonsense-mediated mRNA decay pathway [11], the phenotypic pathogenicity of missense mutations was impossible to predict. Here, in order to understand the biochemical effects of the missense mutations we have expressed mutant proteins in cultured human fibroblasts and COS-7 cells and found that all mutations result in misfolding of the enzyme. As a consequence, it is abnormally glycosylated, and is not targeted to the lysosome, but retained in the endoplasmic reticulum (ER).

## Results

### Expression, processing and enzymatic activity of HGSNAT mutants

**Figure 1:**
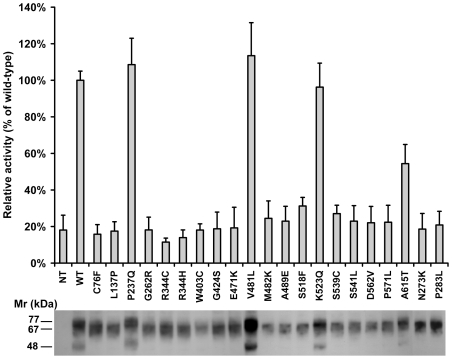
Enzymatic activity and expression of HGSNAT mutants. COS-7 cells were harvested 42 h after transfection with HGSNAT-TAP plasmids bearing missense mutations. Cell homogenates were (A) assayed for N-acetyltransferase activity and (B) analyzed by Western blot using anti-CBP antibody as described in Materials and Methods. A. N-acetyltransferase activity is shown as a fraction of the activity measured in the cells transfected with the wild-type HGSNAT-TAP plasmid. Values represent means ± S.D. of three independent experiments. B. Blot shows a representative image of triplicate experiments.

The effect of HGSNAT mutations on the enzyme biogenesis and catalytic activity was studied by the transient expression of the mutant cDNA. Mutations were generated by site directed mutagenesis in the pCTAP-HGSNAT construct that expresses human HGSNAT with a C-terminal tandem affinity purification (TAP) tag consisting of a high affinity streptavidin-binding peptide (SBP) and a calmodulin-binding peptide (CBP) to allow purification of the recombinant protein using successively-applied streptavidin-resin and calmodulin-resin affinity purification steps or its detection with anti-CBP antibodies [14], [15]. In preliminary experiments we have ensured that a human wild-type HGSNAT carrying a TAP tag on its C-terminus had a lysosomal localization and showed catalytic activity similar to that of the untagged enzyme (not shown). The sequences of the constructs were verified to ensure the correct introduction of mutations. In total, 21 constructs bearing nucleotide changes identified in patients were transfected in COS-7 cells and assayed for N-acetyltransferase activity (Figure 1). All tested cells showed similar endogenous lysosomal β-hexosaminidase activity (not shown) but drastically different levels of the N-acetyltransferase activity. The four polymorphisms (P237Q, V481L, K523Q and A615T) displayed activity similar to that of the wild-type enzyme, whereas the activity of the rest of the mutants was below the detection level. Kinetics studies were further conducted using partially purified enzyme to determine if the P237Q and V481L mutants showing full enzymatic activity would have a different affinity for the substrate, however their K_m_ and V_max_ values were similar to those of the wild-type (Figure S1).

**Figure 2:**
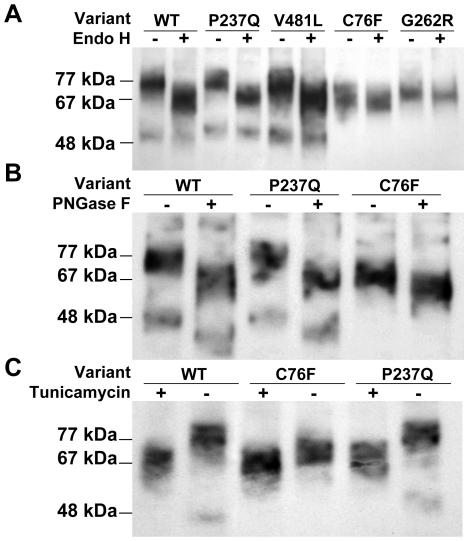
Deglycosylation of HGSNAT by endoglycosidase H, PNGase F and tunicamycin treatment. COS-7 cells expressing either the wild-type HGSNAT or the protein containing C76F, P237Q, G262R or V481L variants were harvested 42 h post-transfection and their homogenates were treated overnight with endoglycosidase H (A) or PNGase F (B). (C) COS-7 cells expressing wild-type HGSNAT and C76F or P237Q variants were cultured for 48 h in the presence or absence of 1 µg/ml of tunicamycin added to the culture medium 5 h after the transfection. The treated and control homogenates were analyzed by Western blot using anti-CBP antibodies as described in Materials and Methods.

**Figure 3:**
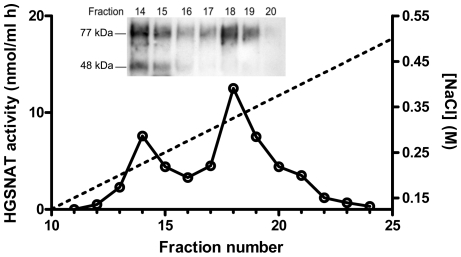
Analysis of the wild-type recombinant HGSNAT by anion-exchange FPLC. COS-7 cells were harvested 42 h after transfection with the wild-type HGSNAT plasmid, solubilized in a buffer containing 0.1% NP-40, applied to an ion-exhange Mono Q HR 5/5 column and eluted by 0-0.5 M NaCl gradient as described in Materials and Methods. Graph shows N-acetyltransferase activity (nmol/hr ml) in the collected fractions. Dashed line represents the NaCl gradient. An aliquot from each fraction was analyzed by Western blot using anti-CBP antibodies (inset).

The expression of HGSNAT mutants was studied by Western blot analysis of cellular homogenates using anti-CBP antibodies (Figure 1B). All mutants were expressed at a level approximately similar to that of the wild-type HGSNAT, but showed a difference in their molecular mass. The four polymorphisms (P237Q, V481L, K523Q and A615T) showed a molecular mass of ∼77 kDa, similar to that of the wild-type enzyme, whereas all missense mutants (C76F, L137P, G262R, N273K, P283L, R344C, R344H, W403C, G424S, E471K, M482K, A489E, S518F, S539C, S541L, D562V, P571L) had a smaller molecular mass of ∼67 kDa. Since HGSNAT is predicted to have 5 potential N-linked glycosylation sites each potentially contributing ∼2 kDa to the size of the mature enzyme [11], this molecular mass difference was consistent with the hypothesis that inactive mutants lacked proper glycosylation. To confirm this, the wild-type enzyme, 2 polymorphic variants and 2 inactive mutants were treated with endoglycosidase H which cleaves immature mannose-rich oligosaccharide chains added to glycoproteins in the ER [16] Analysis by Western blot (Figure 2) showed that upon deglycosylation the molecular mass of the wild-type enzyme and polymorphic mutants was reduced to 67 kDa, whereas the inactive mutants displayed only a subtle shift in their position on the gel, indicating that they already lacked most of the glycans. The homogenates were also treated with the peptide: N-Glycosidase F (PNGase F) which fully removes all sugars from mature glycoproteins (Figure 2B). In addition to performing enzymatic deglycosylation of cell homogenates we treated the COS-7 cells transiently expressing the wild-type enzyme, a polymorphic and an inactive mutant with tunicamycin, an inhibitor of N-acetylglucosamine transferase that blocks glycosylation of newly-synthesized proteins (Figure 2C). Results of both experiments were consistent with those obtained with Endoglycosidase H. Upon treatment with tunicamycin or PNGase F the size of the wild type HGSNAT and the enzyme containing the polymorphism was reduced by approximately ∼10 kDa whereas the inactive mutant displayed only a minor change in electrophoretic mobility. Western blot analysis also revealed that homogenates of cells transfected with both wild-type HGSNAT and polymorphic mutants, but not of untransfected cells or cells transfected with inactive mutants contained an immunoreactive protein with a molecular mass of ∼48 kDa. Since this form of HGSNAT contains the C-terminal CBP cross-reacting with the antibodies we estimated that it corresponds to the HGSNAT fragment missing ∼300 amino acids on the N-terminus. It seems that the appearance of this form is associated with the proper glycosylation and lysosomal targeting of HGSNAT, but further studies are necessary to conclude whether it represents a product of intralysosomal maturation (with the N-terminal chain not being detected by our antibodies) or a degradation product. On the other hand, the 77-kDa form of HGSNAT separated by FPLC anion-exchange chromatography showed full enzymatic activity (Figure 3).

### Subcellular localization of HGSNAT mutants

The effects of missense mutations on the subcellular localization of the enzyme were studied by confocal immunofluorescence microscopy. Immortalized human skin fibroblasts from a normal control were transfected with constructs expressing each of the missense mutations. The cells were allowed to express the mutant and polymorphic enzymes for 42 hours and were then fixed by paraformaldehyde. To identify the lysosomal-late endosomal compartment, prior to fixation the cells were treated with LysoTracker Red DND-99 dye. After fixation the cells were permeabilized with Triton X-100 and probed with anti-CBP antibodies to localize the HGSNAT and with antibodies against the ER marker calnexin and lysosomal marker LAMP-2.

**Figure 4:**
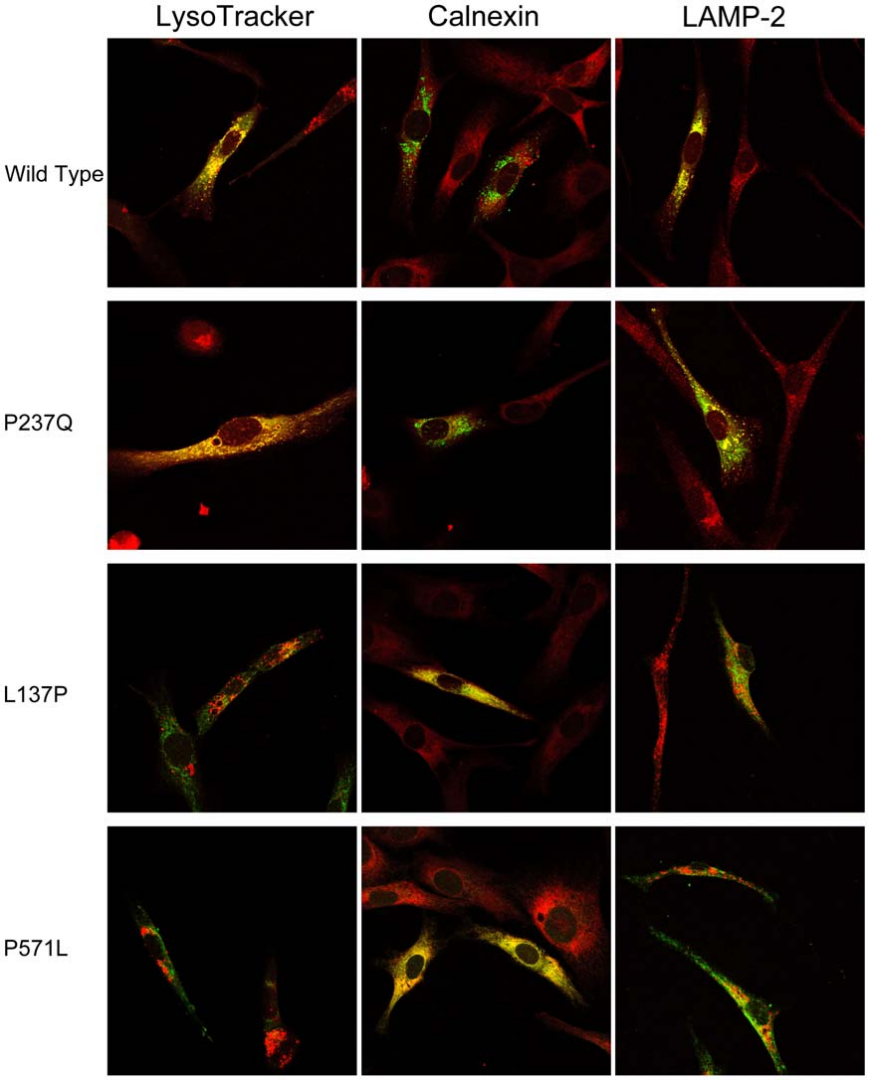
Localization of HGSNAT mutants expressed in cultured human skin fibroblasts by immunofluorescence microscopy. The cells transfected with wild-type or mutant HGSNAT-TAP constructs as indicated were fixed and stained with either mouse monoclonal anti-LAMP-2 antibodies, Lysotracker Red DND-99 or mouse monoclonal anti-calnexin antibodies (red) and rabbit polyclonal anti-CBP antibodies (green) as indicated. Slides were studied on a Zeiss LSM510 inverted confocal microscope. Magnification 630x. Panels show representative images illustrating co-localization of anti-CBP antibodies (green) and lysosomal and ER markers (red) for the wild-type HGSNAT, active enzyme containing P237Q polymorphism and inactive L137P and P571L mutants. From 10 to 15 cells all showing similar localization patterns were studied for each variant. See Figure S2 for the data on other mutants.

Distinct punctate staining characteristic of lysosomal targeting of the protein was evident for the recombinant wild-type enzyme and all active mutants. Accordingly, both wild-type recombinant HGSNAT and polymorphic mutants almost completely co-localized with the lysosomal markers LysoTracker Red or LAMP-2 (representative data are shown in Figure 4). In contrast, the inactive mutants exhibited a diffuse pattern throughout the cell and were co-localized not with the lysosomal markers but with the ER marker calnexin (representative data are shown in Figure 4, see Figure S2 for the data on all mutants). Partial ER localization (typically 2–5% of the total, not shown) was also observed for the wild-type recombinant and active mutants and most likely represented a pool of the enzyme processed in the ER on its way to the lysosomes.

### Glucosamine-mediated refolding of inactive HGSNAT mutants

**Figure 5:**
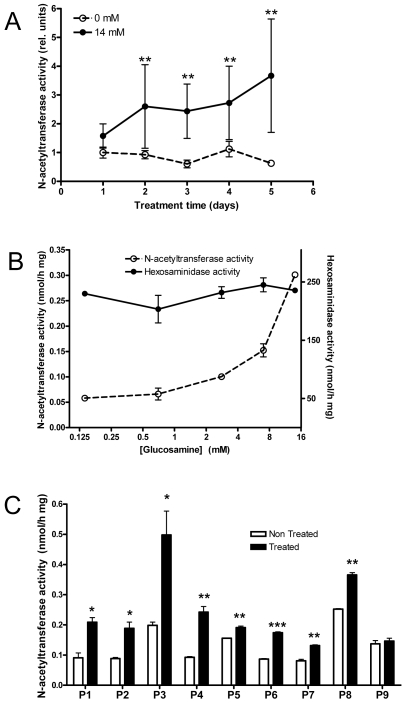
Partial refolding of HGSNAT mutants by glucosamine. A. Fifty percent confluent immortalized skin fibroblasts from a MPS IIIC patient homozygous for N273K mutation [17] were cultured in the presence or absence of 14 mM D-(+)-glucosamine hydrochloride. Medium was replaced every day and at the indicated time intervals cells were harvested and assayed for N-acetyltransferase activity. N-acetyltransferase activity is shown as a fraction of that measured in non-treated cells after 24 h of culturing. Data show mean values and standard error of 2 independent experiments. ** Significantly different (p<0.01) from non-treated cells according to repeated measurements ANOVA. B. Same cells were cultured in the presence of increasing glucosamine concentrations (0–14 mM) for 5 days, harvested and assayed for N-acetyltransferase activity or β-hexosaminidase activity. Data show mean values and standard error of 2 independent experiments. C. Primary skin fibroblasts of the MPS IIIC patients carrying the missense HGSNAT mutations: L137P/S518F (P1), P283L/R344C (P3), S518F/S518F (P4), N273K/N273K (P6), R344C/R344C (P7), S518F/S518F (P8) and E471K/D562V (P9) or a missense mutation in combination with a splice site (S541L/c.234+1G>A; P2) or nonsense (R344H/R384X, P5) mutation were cultured for 2 (P1, P2, P5, P6, P8, P9) or 3 (P3, P4, P7) days in the absence (open bars) or presence (filled bars) of 7 mM (P3-P9) or 14 mM (P1, P2) glucosamine, harvested and assayed for N-acetyltransferase activity. Data show mean values and standard error of 2 independent experiments. The residual N-acetyltransferase activity detectable in untreated cells most likely represents background chemical or enzymatic reactions occurring with the substrate in the presence of cell homogenates. Significantly (*, p<0.05; **, p<0.01; ***, p<0.001) different from non-treated cells according to non-parametric t-test.

Since all the data were consistent with general folding defects and retention in the ER compartment of the HGSNAT mutants (C76F, L137P, G262R, N273K, P283L, R344C, R344H, W403C, G424S, E471K, M482K, A489E, S518F, S539C, S541L, D562V, P571L) we further tested whether competitive inhibitors of the enzyme that mimic the substrate binding in the active site would help to fold the enzyme in the ER so it can be properly modified and exported to the lysosome. We first tested several potential inhibitors of HGSNAT that were not expected to be highly toxic for the cultured cells and found that one of them, D-(+)-glucosamine hydrochloride, was a competitive inhibitor of the enzyme with a K_I_ of 0.28 mM close to the K_M_ value for the 4MU-βGlcN substrate (Figure S3). To establish the optimal concentration of the inhibitor in the culture medium and the length of the treatment, we have used the immortalized skin fibroblasts of previously reported MPS IIIC patient homozygous for the N273K mutation that causes misfolding of the enzyme (Figure 1 and Figure 4), since they were readily available in our lab [17]. Immortalized fibroblasts from a patient homozygous for a splice site mutation (c.1726+1G>A) [11] and normal immortalized fibroblasts were used as controls. We found that the treatment of fibroblasts from a patient homozygous for the N273K mutation with 14 mM glucosamine results in the progressive increase of N-acetyltransferase activity in cell homogenates which reaches 3 fold induction after 5 days of treatment (Figure 5A). The N-acetyltransferase activity in the normal cells and in the cells of the MPS IIIC patient homozygous for the splice site mutation c.1726+1G>A as well as the control β-hexosaminidase activity in all cell lines were not increased upon the treatment (not shown) suggesting that the observed induction of N-acetyltransferase activity was caused by rescuing the N273K mutant but not by the general induction of lysosomal enzymes. When the cells of the patient affected with the N273K mutation were treated for 5 days with increasing concentrations of glucosamine (Figure 5B) we found that the effect was proportional to the concentration of the inhibitor. At the glucosamine concentration of 14 mM (∼50x K_I_) the N-acetyltransferase activity in the treated cells was 0.3 nmol/h mg which corresponds to ∼7% of the average activity in normal control fibroblasts, but further increase of glucosamine concentration resulted in inhibition of cell growth. To understand whether similar effects could be also observed for other missense mutants the available primary cultures of skin fibroblasts from 9 MPS IIIC patients carrying missense HGSNAT mutations or a missense mutation in combination with a splice site or nonsense mutation were treated with glucosamine and assayed for N-acetyltransferase activity. Nine missense mutations were studied altogether (Figure 5C; patient genotypes are listed in the figure legend). Although the maximal effect for different cell lines was achieved at different concentrations of glucosamine and at different treatment time (not shown), for 8 of 9 cell lines a statistically significant increase in the activity was observed (Figure 5C), suggesting that some of the mutants were partially stabilized by the glucosamine, correctly processed and targeted to the lysosomal compartment.

## Discussion

**Figure 6:**
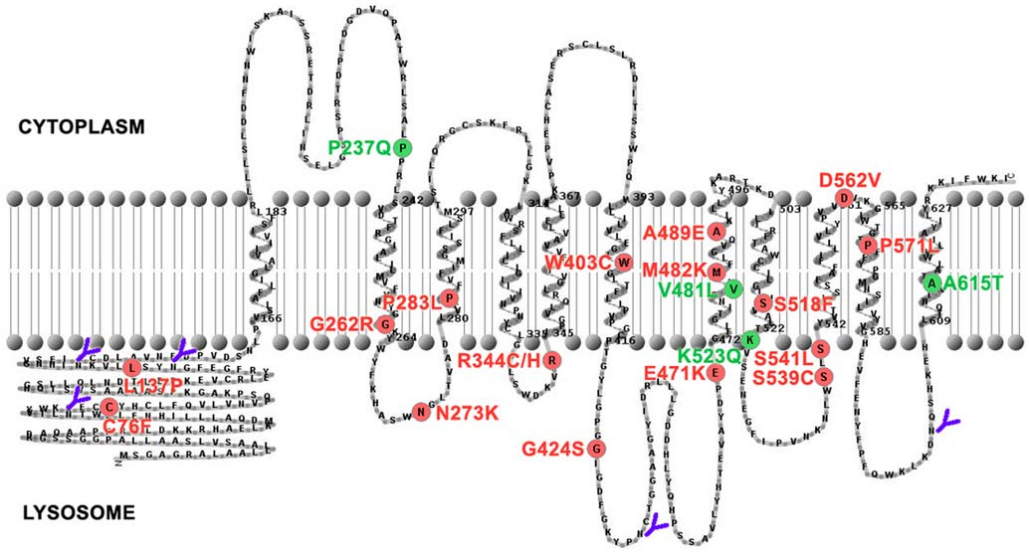
Distribution of missense mutations in HGSNAT protein. Visual representation of HGSNAT membrane topology was created using the TMRPres2D software [26]. The deduced amino acid sequence of HGSNAT predicts 11 transmembrane domains and five potential N-glycosylation sites oriented towards the lysosomal lumen (shown in blue). Mutations that result in production of misfolded proteins are shown in red. Polymorphisms are shown in green. Figure was adapted from our previous work [13].

The current work provides an explanation for the severe phenotype of MPS IIIC patients carrying missense mutations in the *HGSNAT* gene. Altogether, we studied the activity and biogenesis of HGSNAT mutants having 21 amino acid substitutions previously identified in MPS IIIC families and affecting 8 of the 11 transmembrane segments of the enzyme as well as its luminal and cytosolic domains (Figure 6). The missense mutations studied in this paper represent all of the currently identified missense HGSNAT mutations. The polymorphisms (P237Q, V481L, K523Q and A615T, shown in green in Figure 6) identified in MPS IIIC only in *cis* either with a splice site mutation or with a missense mutation [11], [13] were previously reported by us to result in catalytically active protein [13]. They were included in the current study because it was unclear whether they still could affect kinetic parameters or targeting of HGSNAT and therefore represent a clinically valuable phenotype. All variants were expressed as TAP-tagged proteins since no Western blotting or cellular immunostaining could be conducted using the patient fibroblasts due to a lack of specific antibodies against human HGSNAT. Our results show that the HGSNAT variants P237Q, V481L, K523Q and A615T are all correctly processed targeted to the lysosome and display full enzymatic activity. We conclude therefore that these four mutations represent rare polymorphisms in the *HGSNAT* gene and do not have clinical significance thus confirming our previous hypothesis [13].

Seventeen mutations (C76F, L137P, G262R, N273K, P283L, R344C, R344H, W403C, G424S, E471K, M482K, A489E, S518F, S539C, S541L, D562V and P571L, shown in red in Figure 6) result in production of misfolded HGSNAT protein that is abnormally glycosylated and not targeted to the lysosome. Seven of these mutations (G262R, P283L, W403C, M482K, A489E, S518F and P571L) are predicted to reside within the highly hydrophobic transmembrane domains of the protein. Four of them (G262R, W403C, M482K, A489E) introduce hydrophilic or charged residues inside the transmembrane domains which usually have a dramatic effect on the folding of the protein [reviewed in 18]. Three other changes (P283L, S518F and P571L) result in replacement of hydrophilic residues for hydrophobic ones that also could destabilize the transmembrane helix.

Six mutations (R344C, R344H, E471K, S539C, S541L and D562V) are found adjacent to the predicted transmembrane domains either on the cytoplasmic (D562V) or on the lumenal (R344C, R344H, E471K, S539C, and S541L) side and 4 mutations (C76F, L137P, N273K, and G424S) reside inside the hydrophilic lumenal domains of the enzyme. In most cases these mutations are predicted to have a drastic effect on protein folding since they involve replacements with amino acids significantly different in hydrophobicity (C76F, D562V, S541L), charge (R344C/H, N273K, E471K) or size (C76F, L137P, G424S). Thus, enzyme folding defects due to missense mutations, together with nonsense-mediated mRNA decay seem to be the major molecular mechanisms underlying MPS IIIC.

For at least 5 of the above changes (N273K, R344C, R344H, S518F and S541L) the active conformation can be stabilized by the competitive inhibitor of HGSNAT glucosamine resulting in part of the enzyme pool being properly processed and targeted to the lysosomes. L137P and P283L mutants may also be stabilized by the glucosamine treatment, however this could not be verified experimentally because in the available patient cell lines they were present together with the responsive mutations S518F and R344C, respectively. Only one cell line carrying E471K and D562V mutations did not show a significant increase in N-acetyltransferase activity in response to glucosamine. Further structural studies are needed to fully understand the difference in the effect of glucosamine on these mutants.

Although the spectrum of mutations in MPS IIIC patients shows substantial heterogeneity, some of the missense mutations have a high frequency within the patient population. Importantly, the two mutations, R344C and S518F, responsive to glucosamine-mediated refolding account for 22.0% and 29.3%, respectively, of the alleles among the probands of Dutch origin [19]. The S518F mutation has also been identified in a patient from Germany, while the R344C change was found in families from France, UK, Germany and Singapore. The responsive mutation R344H was found in 4 families from Eastern and Northern Europe (2 from Poland, one from Czech Republic and one from Finland) and the responsive mutation S541L was reported in 4 families from France, Ireland, Poland and Portugal. In general, the vast majority of patients is affected with at least one missense mutation interfering with the proper folding of the enzyme that could be partially rescued by the treatment of the cells with the competitive inhibitor of HGSNAT, glucosamine. We believe this makes MPS IIIC a good candidate for enzyme enhancement therapy [reviewed in 20], [21], where active site-specific inhibitors are used as pharmacological chaperones to modify the conformation of the mutant lysosomal enzymes usually retained and degraded in the ER in order to increase the level of the residual activity to a point sufficient to reverse the clinical phenotype. Together with inhibitors of heparan sulfate synthesis, pharmacological chaperones could potentially reduce storage of this polymer in the central nervous system to levels sufficient to stop neuronal death and reverse inflammation.

## Materials and Methods

The current study was conducted with ethics approval from the review board of CHU Ste-Justine, University of Montreal.

### Generation of expression constructs and site-directed mutagenesis

The wild-type HGSNAT-TAP plasmid was obtained by subcloning the HGSNAT 1992 bp coding sequence into pCTAP vector (Stratagene). Briefly, a 3′ part of human pCMV-Script construct [11] missing the stop codon was amplified by PCR using primers 76-Cla-F 5′-TTG CTC TTA TAC TCA TGG TCT TTG TCA-3′ and TM76-R4 5′-ATA TGT CGA CGA GCC ATC CGA TTT TCC-3′ and then used to replace Cla I - Sal I segment of the same construct prior to subcloning into pCTAP using Hind III – Sal I sites. Missense mutants were constructed using QuikChange Lightning kit (Stratagene) with HGSNAT-TAP as a matrix (see Table S1 for primers). All primers were designed using QuikChange Primer Design Program (http://www.stratagene.com/qcprimerdesign). For all constructs the coding sequence included an extra 84 base pairs on their 5′ end (encoding 28 amino acids) as the sequence first described for HGSNAT gene [11], but for clarity the nomenclature used reflects a unified numbering system based on the sequence of GenBank entries NM_152419.2 and NG_009552.1 as in Fan et al. [12], Ruijter et al. [19], Fedele et al. [22] and Feldhammer et al. [13].

### Cell culture and transfection

Skin fibroblast lines of MPS IIIC patients, obtained with a written informed consent [11], and COS-7 cells (ATCC) were cultured in Eagles's minimal essential medium supplemented with 10% (v/v) fetal calf serum (Wisent). Transfections were carried out using polyethyleneimine 25,000 (PEI, Polysciences inc.) or Lipofectamine LTX (Invitrogen). PEI was dissolved in water at 1 mg/ml and pH was adjusted to 6.8 with HCl. For transfection, a mixture of 2 µg DNA and 8 µg PEI in 0.4 ml of serum free medium was incubated for 15 min at room temperature and added to ∼70% confluent cells growing in a 10 cm dish. The transfection media was replaced with growth media 18 h later. Lipofectamine LTX was used as described in the manufacturer's protocol. Patients (N273K and c.1726+1G>A) and control fibroblasts immortalized by transfection with retroviral vectors expressing the type 16 human papilloma virus E7 gene and the catalytic component of human telomerase were described before [17].

### Glucosamine-mediated refolding

Patient and control skin fibroblasts were cultured as described above in growth media supplemented with various concentrations of D-(+)-glucosamine hydrochloride (Sigma G4875). Medium was replaced every day and at specified time cells were harvested and assayed for N-acetyltransferase activity and β-hexosaminidase activity as described below.

### Enzyme assays

N-acetyltransferase enzymatic activity was measured using fluorogenic substrate, 4-methylumbelliferyl *β*-D-glucosaminide (4MU-βGlcN, Moscerdam, Rotterdam, The Netherlands) as previously described by He et al. [23]. The reaction mixture containing 5 µl of cell homogenate, 5 µl of 6 mM acetyl-CoA and 5 µl of 3 mM 4MU-βGlcN in McIlvain buffer (100 mM sodium citrate, 200 mM sodium phosphate, pH 5.7) was incubated at 37°C for 3–18 h. The reaction was terminated by adding 1.98 ml of 0.5 M Na_2_CO_3_/NaHCO_3_, pH 10.7, and fluorescence was measured and used to calculate the specific activity. Lysosomal β-hexosaminidase activity was measured as previously described [24]. Protein concentration was measured according to the method of Bradford [25] using a commercially available reagent (BioRad).

### Deglycosylation of HGSNAT

To remove N-linked glycans from HGSNAT, cell homogenates were treated with recombinant endoglycosidase H, or peptide: N-Glycosidase F (Endo H; PNGase F New England Biolabs). Briefly, the mix consisted of 10 µg of cell homogenate in 25 mM sodium phosphate buffer, pH 7.5, to which 1 µl (500 U) of concentrated Endo H or PNGase F was added before incubating at 37°C overnight and analysis by Western blot.

To inhibit glycosylation of newly-synthesized HGSNAT COS-7 cells transfected with plasmids coding for the wild-type enzyme and the C76F or P237Q variants were treated with 1 µg/ml of tunicamycin (Sigma) added 5 h after transfection and allowed to express the recombinant protein for 48 h in the presence of drug. Media and drug were changed after 24 h and the homogenates were analyzed by Western blot as described below.

### Western blotting

Cell homogenates were sonicated and boiled in LDS sample buffer (Invitrogen) in the presence of 25 mM DTT. Proteins were resolved by SDS-polyacrylamide gel electrophoresis using NuPAGE 4–12% Bis-Tris gels (Invitrogen) and electrotransferred to PVDF membrane. Detection of TAP-tagged N-acetyltransferase protein was performed using anti-calmodulin binding peptide epitope tag (CBP) rabbit antibodies (Immunology Consultants Laboratory, dilution 1∶30,000) and the Amersham ECL Western Blotting Detection Reagents (GE Healthcare) in accordance with the manufacturer's protocol.

### Analysis of the wild-type recombinant HGSNAT by anion-exchange FPLC

COS-7 cells were harvested 42 h after transfection with the wild-type HGSNAT plasmid and suspended in lysis buffer (10 mM Tris-HCl, pH 7.5, 0.1% NP-40, 1 mM PMSF and Sigma P8340 protease inhibitor cocktail at 10 µl per 1 ml of cell suspension). The homogenate was sonicated, gently shaked at 4°C for 2 h and centrifuged at 13,000 rpm for 30 min. One ml of the supernatant containing 8 mg of total protein was applied to an ion-exhange Mono Q HR 5/5 column equilibrated with 10 mM Tris buffer, pH 8.2. The column was washed with 4 ml of the same buffer and then eluted using a 20 mL gradient of NaCl (0–0.5 M) at a flow rate of 0.5 ml/min. One ml fractions were collected and assayed for N-acetyltransferase activity. Thirty µl aliquots from each fraction were analyzed by SDS-PAGE and Western blot using anti-CBP antibodies as described above.

### Confocal immunofluorescence microscopy

Immortalized control human skin fibroblasts were transfected with HGSNAT-TAP or plasmids coding for the HGSNAT mutants using Lipofectamine LTX (Invitrogen) as described in the manufacturer's protocol. Forty-two hours post-transfection cells were incubated for 1 hour with 1 µM Lysotracker Red DND-99 (Invitrogen) and then washed with ice-cold PBS. Cells were fixed with 4% paraformaldehyde, 4% sucrose in PBS for 5 min, and then rinsed 3 times with PBS. Cells were permeabilized by 0.25% Triton X-100 for 10 min and blocked for 1 h in 3% horse serum and 0.1% Triton X-100. Cells were either co-stained with rabbit anti-CBP (Immunology Consultants Laboratory; 1∶400) and mouse monoclonal anti-calnexin (Millipore; 1∶250) antibodies in 3% horse serum, with anti-CBP antibodies and mouse monoclonal antibodies against human LAMP-2 (Developmental Studies Hybridoma Bank; 1∶150), or with anti-CBP antibodies and Lysotracker Red DND-99. Cells were then counterstained with Oregon Green 488-conjugated anti-rabbit IgG antibodies or Texas-Red-conjugated goat anti-mouse antibodies (Molecular Probes; 1∶1000). Slides were studied on a Zeiss LSM510 inverted confocal microscope (Zeiss). Images were processed using the LSM image browser software (Zeiss) and Photoshop (Adobe).

## Supporting Information

Figure S1 Lineweaver-Burk plot of substrate dependance for partially purified HGSNAT wild-type and P237Q and V481L mutants. COS-7 cells were harvested 42 hrs after transfection with the wild-type or mutant HGSNAT plasmids and suspended in lysis buffer (40 mM Tris-HCl, 300 mM KCl, pH 7.5, 0.1% NP-40, 1 mM PMSF and Sigma P8340 protease inhibitor cocktail at 10 µl per 1 ml of cell suspension). The homogenate was sonicated, gently shaked at 4°C for 2 h and centrifuged at 13,000 rpm for 30 min. The supernatant was first passed through an avidin-agarose column (Sigma A9207) then affinity purification of TAP-tagged HGSNAT was performed using streptavidin resin (Stratagene) according to the manufacturer's protocol. N-acetyltransferase activity was assayed as described in Material and Methods using 0.05 to 2.0 mM 4MU-βGlcN and 18 h incubation time. KM and VMAX values for all 3 enzymes were similar within the statistical error.
(0.07 MB PPT)

Figure S2 Localization of HGSNAT mutants expressed in cultured human skin fibroblasts by immunofluorescence microscopy. The cells transfected with wild-type or mutant HGSNAT-TAP constructs as indicated were fixed and stained with either mouse monoclonal anti-LAMP-2 antibodies, Lysotracker Red DND-99 or mouse monoclonal anti-calnexin antibodies (red) and rabbit polyclonal anti-CBP antibodies (green) as indicated. Slides were studied on a Zeiss LSM510 inverted confocal microscope. Magnification 630x. Panels show representative images showing co-localization of anti-CBP antibodies (green) and lysosomal and ER markers (red) for the active enzyme containing polymorphisms and all inactive mutants.
(5.80 MB PPT)

Figure S3 Dixon plot showing the inhibition of HGSNAT by glucosamine. COS-7 cells were harvested 42 hrs after transfection with the wild-type HGSNAT plasmid and N-acetyltransferase activity was measured in the homogenates for 3 h at 37°C in the presence of 2 mM AcCoA, 0.0375 to 1.5 mM 4MU-βGlcN and 0 to 2 mM D-(+)-glucosamine hydrochloride.
(0.06 MB PPT)

Table S1 Primers for site-directed mutagenesis of HGSNAT-TAP plasmid.
(0.04 MB DOC)
