# Supplementary figures and images for: Protein Misfolding as an Underlying Molecular Defect in Mucopolysaccharidosis III Type C
Source: PLoS One. 2009 Oct 13;4(10):e7434. doi: 10.1371/journal.pone.0007434 (PMC2757673; doi:10.1371/journal.pone.0007434)

## Slide 1
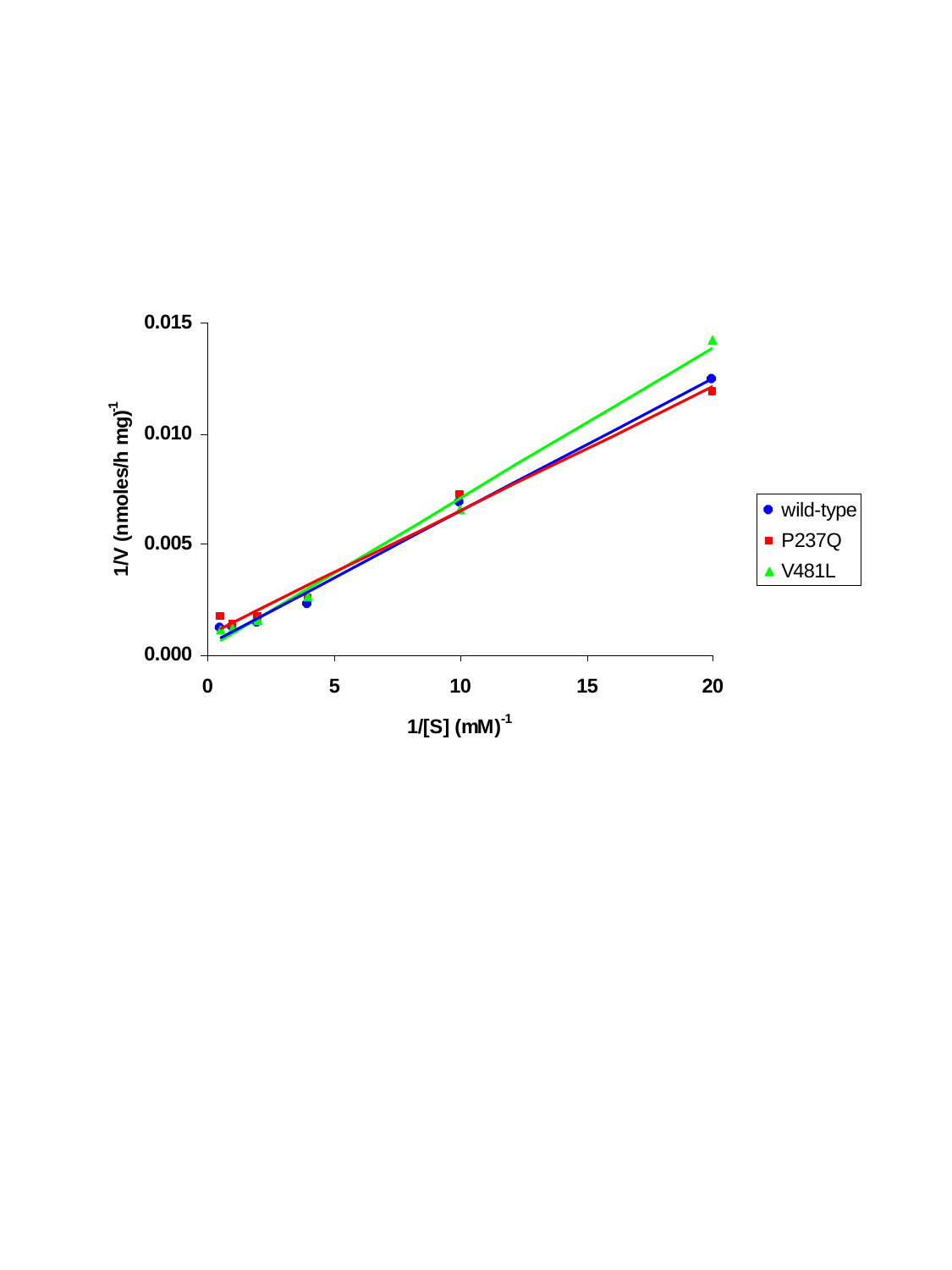

Supplement: Figure S1 — Lineweaver-Burk plot of substrate dependance for partially purified HGSNAT wild-type and P237Q and V481L mutants. COS-7 cells were harvested 42 hrs after transfection with the wild-type or mutant HGSNAT plasmids and suspended in lysis buffer (40 mM Tris-HCl, 300 mM KCl, pH 7.5, 0.1% NP-40, 1 mM PMSF and Sigma P8340 protease inhibitor cocktail at 10 µl per 1 ml of cell suspension). The homogenate was sonicated, gently shaked at 4°C for 2 h and centrifuged at 13,000 rpm for 30 min. The supernatant was first passed through an avidin-agarose column (Sigma A9207) then affinity purification of TAP-tagged HGSNAT was performed using streptavidin resin (Stratagene) according to the manufacturer's protocol. N-acetyltransferase activity was assayed as described in Material and Methods using 0.05 to 2.0 mM 4MU-βGlcN and 18 h incubation time. KM and VMAX values for all 3 enzymes were similar within the statistical error. (0.07 MB PPT) [file pone.0007434.s001.ppt]

## Slide 1
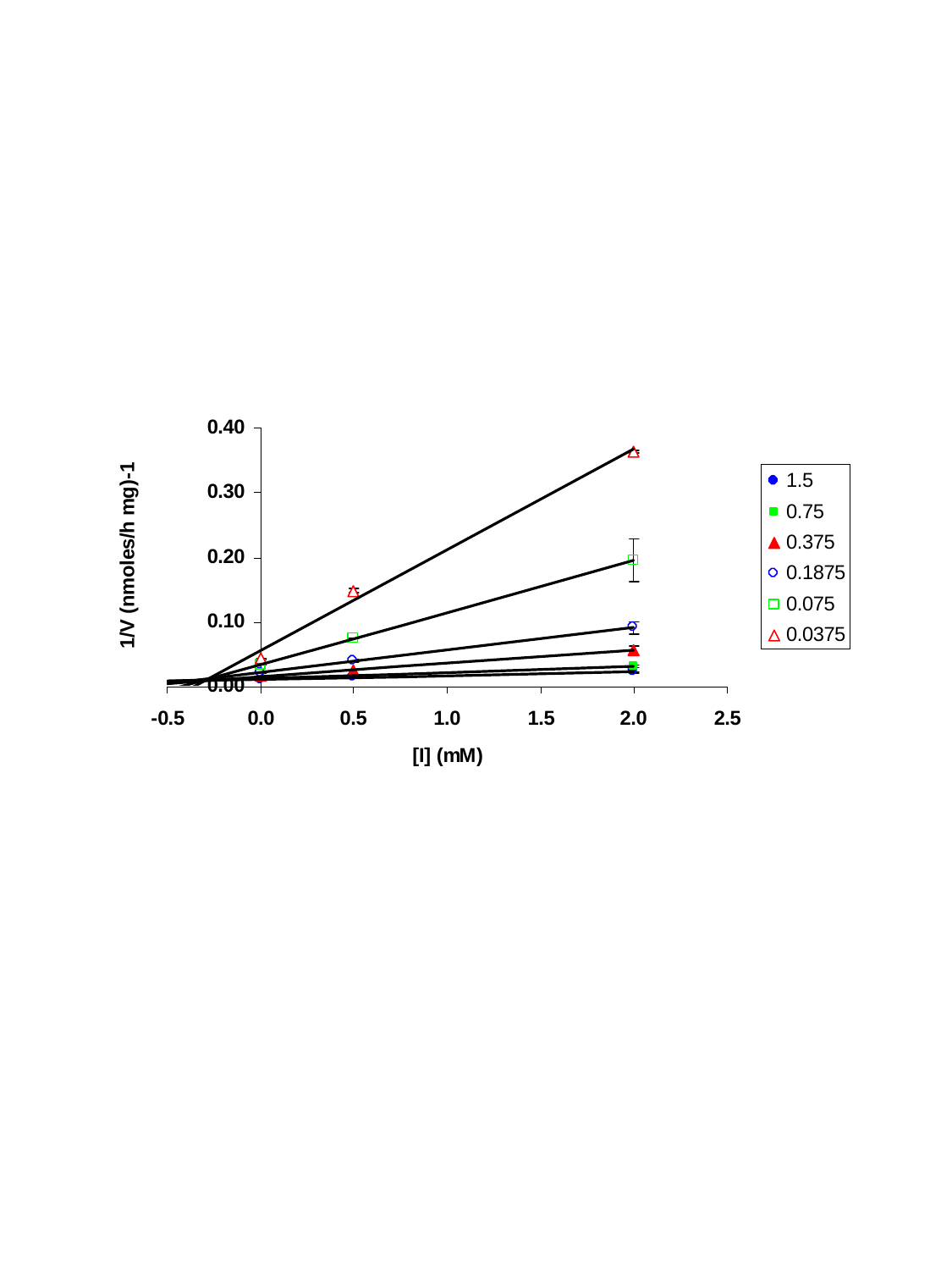

Supplement: Figure S3 — Dixon plot showing the inhibition of HGSNAT by glucosamine. COS-7 cells were harvested 42 hrs after transfection with the wild-type HGSNAT plasmid and N-acetyltransferase activity was measured in the homogenates for 3 h at 37°C in the presence of 2 mM AcCoA, 0.0375 to 1.5 mM 4MU-βGlcN and 0 to 2 mM D-(+)-glucosamine hydrochloride. (0.06 MB PPT) [file pone.0007434.s003.ppt]
