# Supplementary material for: Protein Misfolding as an Underlying Molecular Defect in Mucopolysaccharidosis III Type C
Source: PLoS One. 2009 Oct 13;4(10):e7434. doi: 10.1371/journal.pone.0007434 (PMC2757673; doi:10.1371/journal.pone.0007434)
Supplement: Figure S2 — Localization of HGSNAT mutants expressed in cultured human skin fibroblasts by immunofluorescence microscopy. The cells transfected with wild-type or mutant HGSNAT-TAP constructs as indicated were fixed and stained with either mouse monoclonal anti-LAMP-2 antibodies, Lysotracker Red DND-99 or mouse monoclonal anti-calnexin antibodies (red) and rabbit polyclonal anti-CBP antibodies (green) as indicated. Slides were studied on a Zeiss LSM510 inverted confocal microscope. Magnification 630x. Panels show representative images showing co-localization of anti-CBP antibodies (green) and lysosomal and ER markers (red) for the active enzyme containing polymorphisms and all inactive mutants. (5.80 MB PPT) [file pone.0007434.s002.ppt]

## Slide 1
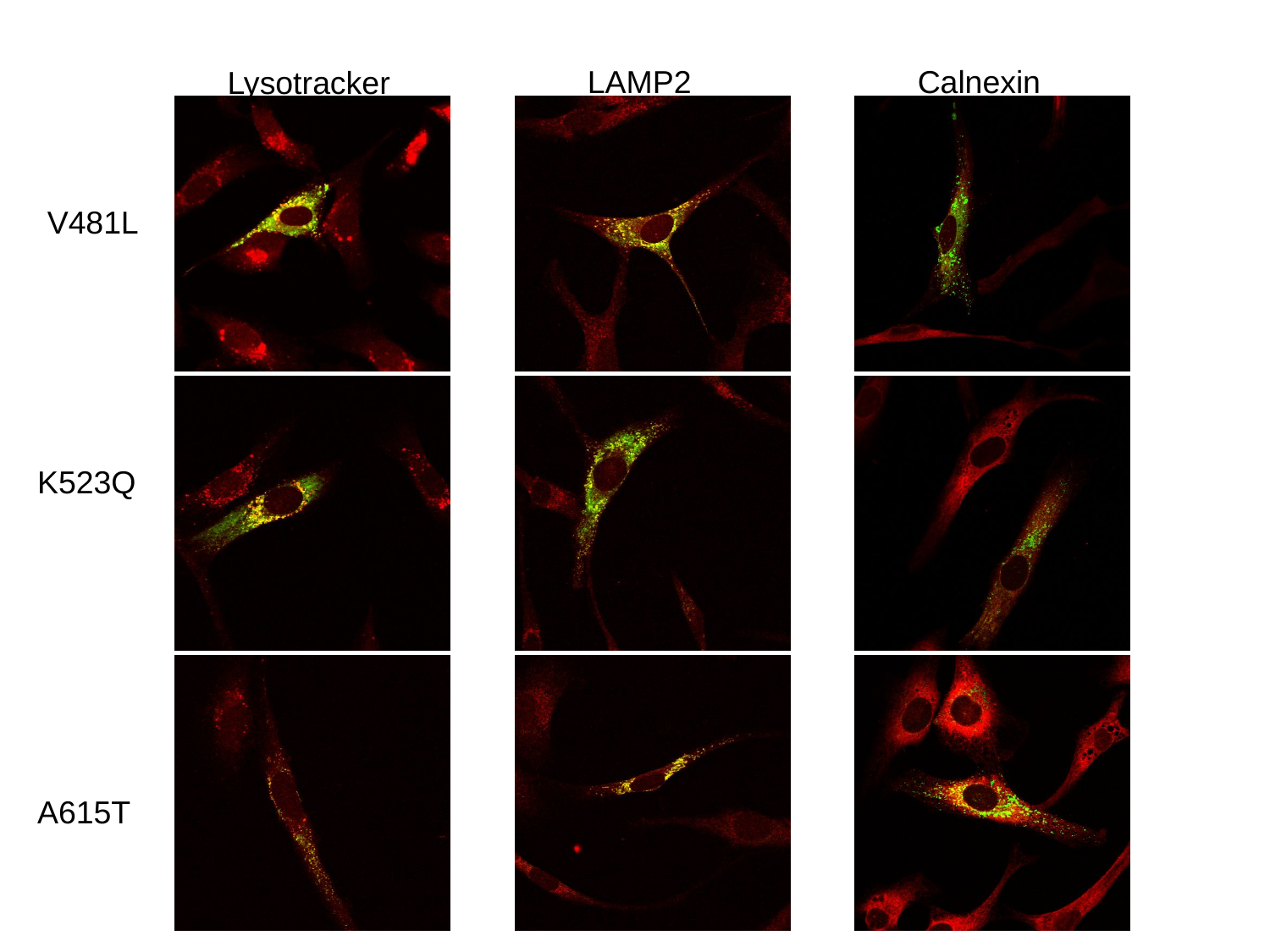

LAMP2
Calnexin
Lysotracker
V481L
K523Q
A615T

## Slide 2
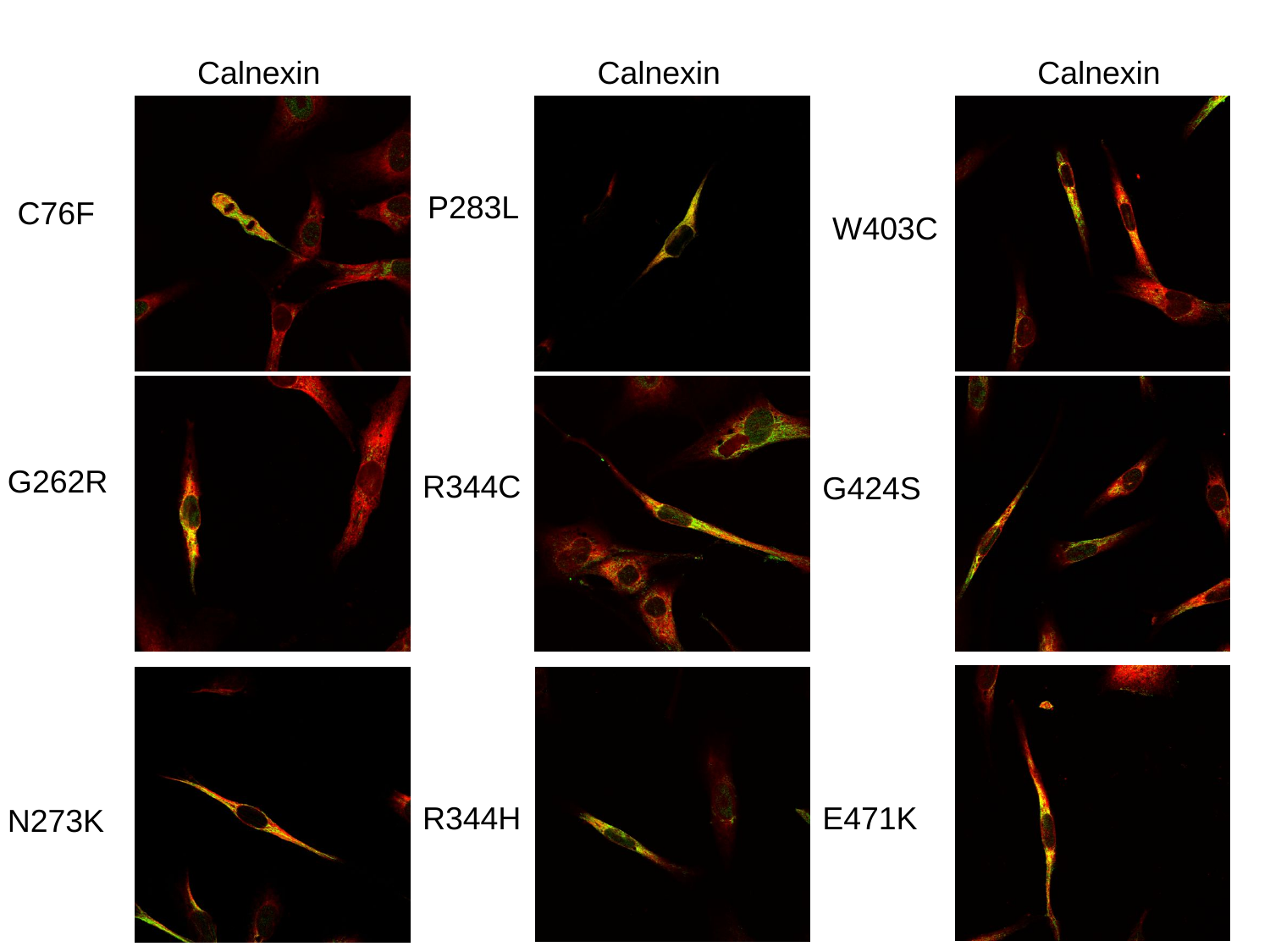

Calnexin
Calnexin
Calnexin
P283L
C76F
W403C
G262R
R344C
G424S
R344H
E471K
N273K

## Slide 3
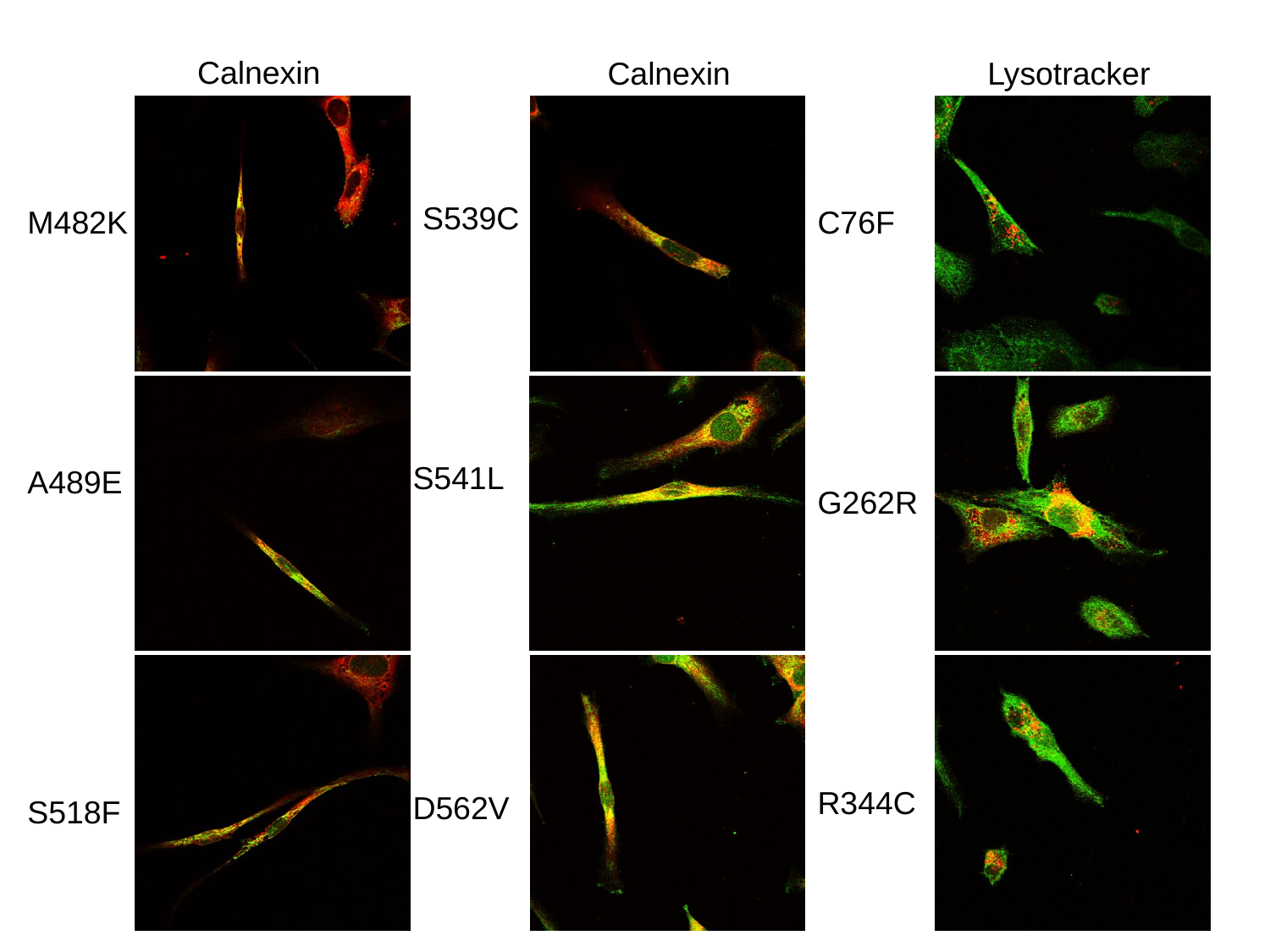

Calnexin
Calnexin
Lysotracker
S539C
M482K
C76F
S541L
A489E
G262R
R344C
D562V
S518F
