# Supplementary material for: Protein Misfolding as an Underlying Molecular Defect in Mucopolysaccharidosis III Type C
Source: PLoS One. 2009 Oct 13;4(10):e7434. doi: 10.1371/journal.pone.0007434 (PMC2757673; doi:10.1371/journal.pone.0007434)
Supplement: Table S1 — Primers for site-directed mutagenesis of HGSNAT-TAP plasmid. (0.04 MB DOC) [file pone.0007434.s004.doc]

| Mutant | Sense Primer | Antisense Primer |
| --- | --- | --- |
| C76F | ctactggaaatctgaatgcttttatcactgcttgtttcagg | cctgaaacaagcagtgataaaagcattcagatttccagtag |
| L137P | tggagaatttggaaactattctcccttggtaaagaacatccataat | attatggatgttctttaccaagggagaatagtttccaaattctcca |
| P237Q | atctgccctgcagccccgcctcc | ggaggcggggctgcagggcagat |
| G262R | tggtctttgtcaattatggaggaagaaaatattggtacttcaaacat | atgtttgaagtaccaatattttcttcctccataattgacaaagacca |
| N273K | tacttcaaacatgcaagttggaaggggctgacagtg | cactgtcagccccttccaacttgcatgtttgaagta |
| P283L | tggctgacctcgtgttcctgtggtttgtatttattatg | cataataaatacaaaccacaggaacacgaggtcagcca |
| R344C | tcttgggacaaggtgtgcattcctggtgtgc | gcacaccaggaatgcacaccttgtcccaaga |
| R344H | cttgggacaaggtgcacattcctggtgtgct | agcacaccaggaatgtgcaccttgtcccaag |
| W403C | gctggaaggcctgtgtctgggcttgacattc | gaatgtcaagcccagacacaggccttccagc |
| G424S | gttatcttggtcctgggagcattggagattttggc | gccaaaatctccaatgctcccaggaccaagataac |
| E471K | ggcctatgaccccaagggcatcctggg | ccggatactggggttcccgtaggaccc |
| V481L | gcaccatcaactccatcttgatggcctttttagga | tcctaaaaaggccatcaagatggagttgatggtgc |
| M482K | gcaccatcaactccatcgtgaaggcctttttagg | cctaaaaaggccttcacgatggagttgatggtgc |
| A489E | atggcctttttaggagttcaggaaggaaaaatactattgtattaca | tgtaatacaatagtatttttccttcctgaactcctaaaaaggccat |
| S518F | ttgtattcttgggctcatttttgttgctctgacgaagg | ccttcgtcagagcaacaaaaatgagcccaagaatacaa |
| K523Q | catttctgttgctctgacgcaggtttctgaaaatgaagg | ccttcattttcagaaacctgcgtcagagcaacagaaatg |
| S539C | ccagtaaacaaaaatctctggtgcctttcgtatgtcactac | gtagtgacatacgaaaggcaccagagatttttgtttactgg |
| S541L | aaaatctctggtcccttttgtatgtcactacgctcag | ctgagcgtagtgacatacaaaagggaccagagatttt |
| D562V | tgtacccagttgtggttgtgaaggggctgtg | cacagccccttcacaaccacaactgggtaca |
| P571L | gctgtggacaggaaccctattcttttatccaggaa | ttcctggataaaagaatagggttcctgtccacagc |
| A615T | ctcagaacatcgtcaccactgccctctgg | ccagagggcagtggtgacgatgttctgag |
